# Biochemical and transcriptomic analyses reveal that critical genes involved in pigment biosynthesis influence leaf color changes in a new sweet osmanthus cultivar ‘Qiannan Guifei’

**DOI:** 10.7717/peerj.12265

**Published:** 2021-10-08

**Authors:** Qi Cui, Junhua Huang, Fan Wu, Dong-ze Li, Liqun Zheng, Guang Hu, Shaoqing Hu, Lu Zhang

**Affiliations:** School of Civil Engineering and Architecture, Zhejiang Sci-Tech University, Hangzhou, Zhejiang, China

**Keywords:** Osmanthus fragrans ‘Qiannan Guifei’, Leaf color, Chlorophyll, Carotenoid, Flavonoid, Anthocyanin, OfMYB genes

## Abstract

**Background:**

*Osmanthus fragrans* (Oleaceae) is one of the most important ornamental plant species in China. Many cultivars with different leaf color phenotypes and good ornamental value have recently been developed. For example, a new cultivar ‘Qiannan Guifei’, presents a rich variety of leaf colors, which change from red to yellow-green and ultimately to green as leaves develop, making this cultivar valuable for landscaping. However, the biochemical characteristics and molecular mechanisms underlying leaf color changes of these phenotypes have not been elucidated. It has been hypothesized that the biosynthesis of different pigments in *O. fragrans* might change during leaf coloration. Here, we analyzed transcriptional changes in genes involved in chlorophyll (Chl), flavonoid, and carotenoid metabolic pathways and identified candidate genes responsible for leaf coloration in the new cultivar ‘Qiannan Guifei’.

**Methods:**

Leaf samples were collected from ‘Qiannan Guifei’ plants at the red (R), yellow-green (YG) and green (G) leaf stages. We compared the different-colored leaves via leaf pigment concentrations, chloroplast ultrastructure, and transcriptomic data. We further analyzed differentially expressed genes (DEGs) involved in the Chl, flavonoid, and carotenoid metabolic pathways. In addition, we used qRT-PCR to validate expression patterns of the DEGs at the three stages.

**Results:**

We found that, compared with those at the G stage, chloroplasts at the R and YG stages were less abundant and presented abnormal morphologies. Pigment analyses revealed that the leaves had higher flavonoid and anthocyanin levels at the R stage but lower Chl and carotenoid concentrations. Similarly, Chl and carotenoid concentrations were lower at the YG stage than at the G stage. By using transcriptomic sequencing, we further identified 61 DEGs involved in the three pigment metabolic pathways. Among these DEGs, seven structural genes (*OfCHS*, *OfCHI*, *OfF3H*, *OfDFR*, *OfANS*, *OfUGT* and*Of3AT*) involved in the flavonoid biosynthesis pathway were expressed at the highest level at the R stage, thereby increasing the biosynthesis of flavonoids, especially anthocyanins. Six putative*OfMYB* genes, including three flavonoid-related activators and three repressors, were also highly expressed at the R stage, suggesting that they might coordinately regulate the accumulation of flavonoids, including anthocyanins. Additionally, expressions of the Chl biosynthesis-related genes *OfHEMA*, *OfCHLG* and *OfCAO* and the carotenoid biosynthesis-related genes *OfHYB* and *OfZEP* were upregulated from the R stage to the G stage, which increased the accumulation of Chl and carotenoids throughout leaf development. In summary, we screened the candidate genes responsible for the leaf color changes of ‘Qiannan Guifei’, improved current understanding of the regulatory mechanisms underlying leaf coloration and provided potential targets for future leaf color improvement in *O. fragrans*.

## Introduction

In many ornamental plants, leaf color is a critical phenotypic trait that affects commercial value. Leaf coloration is controlled mainly by three types of pigments: flavonoids (including anthocyanins), chlorophyll (Chl), and carotenoids. Flavonoids, particularly anthocyanins, are the major source of color in flowers, providing orange, red, pink, and blue colorations. In addition, anthocyanins present in vegetative tissues contribute to the formation of purple and red leaves. The genetic and biochemical basis associated with anthocyanin metabolism have been extensively studied in arabidopsis (*Arabidopsis thaliana*) (Misyura, Colasanti & Rothstein, 2013; Broeckling et al., 2016), petunia (*Petunia hybrida*) (Berenschot & Quecini, 2014; Shaipulah et al., 2016; Naing et al., 2020), and maize (*Zea mays*) (Harakotr et al., 2016; Ramirez et al., 2018). A group of structural genes that play critical roles in the anthocyanin biosynthesis pathway have been identified. These genes can be classified into two categories: those involved in the early anthocyanin biosynthesis pathway, including *phenylalanine ammonia-lyase* (*PAL*), *cinnamate-4-hydroxylase* (*C4H*), *4-coumarate-CoA ligase* (*4CL*), *chalcone synthase* (*CHS*), *chalcone isomerase* (*CHI*), and *flavonoid 3-hydroxylase* (*F3H*), and those involved in the late biosynthesis pathway, including *dihydroflavonol 4-reductase* (*DFR*), *anthocyanidin synthase* (*ANS*), *UDP-glucosyl transferase* (*UGT*), and *anthocyanidin 3-O-glucoside 6″-O-acyltransferase* (*3AT*) (Dick et al., 2011). These structural genes can be effectively regulated by three kinds of transcription factors (TFs), namely, R2R3-MYB, basic helix-loop-helix (bHLH), and WD40 repeat proteins (Feller et al., 2011).

There have been many reports on the genetic basis of red or purple leaf coloration in ornamental plant species and cultivars. For example, previous studies examined the gene expression of structural genes associated with anthocyanin biosynthesis in purple leaves of tea (*Camellia sinensis*) cultivars and found that anthocyanin accumulation is primary affected by *CsANS* and *CsUGT*, whose activity is regulated by *CsAN1* (a member of the *R2R3-MYB* family), *CsGL3* and *CsEGL3* (members of the *bHLH* family) (Kerio et al., 2012; Sun et al., 2016). Jin et al. (2018) elucidated the transcriptional regulation of anthocyanin pigmentation in ornamental cabbage (*Brassica oleracea*), finding that *BoPAP2* (*R2R3-MYB*), *BoMYC1.2* (*bHLH*), and *BoTTG1* (*WD40*) positively controlled anthocyanin production in purple-red leaves of the rosette. Similarly, Kanemaki et al. (2018) found that ectopic expression of an *R2R3-MYB* gene from toad lily (*Tricyrtis* spp.) resulted in the formation of deep red leaves in transgenic geranium (*Pelargonium crispum*) plants.

It has been demonstrated that the chlorotic-leaf phenotype is closely associated with Chl metabolism in plants (Rodrguez et al., 2013). Glutamyl-tRNA reductase (HEMA) catalyzes the initial enzymatic step during tetrapyrrole biosynthesis in chloroplasts, which promotes Chl production. The Chl biosynthesis branch initiates when magnesium (Mg^2+^) is inserted into protoporphyrin IX *via* Mg-chelatase. Mg-chelatase contains three subunits: magnesium chelatase subunit D (CHLD), CHLH, and CHLI (Kumar & Söll, 2000). The entire Chl biosynthetic process is composed of three phases: biosynthesis of Chl a, interconversion between Chl a and Chl b, and degradation of Chl a. The critical genes involved in Chl a biosynthesis are *protochlorophyllide reductase* (*POR*), *Divinyl chlorophyllide a 8-vinyl-reductase* (*DVR*), and *chlorophyll synthase* (*CHLG*); *chlorophyllide a oxygenase* (*CAO*) is the most important gene in Chl b biosynthesis; and *chlorophyll hydrolase* (*CLH*), *pheophorbide an oxygenase* (*PAO*), *chlorophyll b reductase* (*NOL/NYC*), and *magnesium dechelatase* (*MCS*) genes play critical roles in the degradation of Chl a (Meier et al., 2011). Mutations in any of Chl metabolism-related genes may contribute to alteration in Chl concentration, thereby generating chlorotic-leaf mutants. Many efforts have been made to elucidate coloration mechanism in these mutants. For example, a barley (*Hordeum vulgare*) mutant displays chlorotic-leaf phenotype due to a lack of HvCAO enzymatic activity (Rudoi & Shcherbakov, 1998). The formation of yellow-striped leaves in an orchid (*Cymbidium sinense*) mutant was found to be caused mainly by increased expression of *CsCLH*, *CsPAO*, and *CsNOL/CsNYC* genes (Zhu et al., 2015a). In addition, studies on the mutants of rice (*Oryza sativa*) (Kusumi et al., 2011) and tomato (*Solanum lycopersicum*) (Barry et al., 2012) showed that chloroplast development also affected the chlorotic-leaf phenotype.

Carotenoids usually contribute to yellow or orange coloration in plant organs (Kim et al., 2012). The carotenoid biosynthetic pathway starts from a critical rate-limiting reaction, which produces phytoene through the catalysis of phytoene synthase (PSY) on two geranylgeranyl diphosphate (GGPP) molecules. Subsequently, the phytoene is converted to red lycopene *via* a series of reactions by phytoene desaturase (PDS), *ζ*-carotene isomerase (Z-ISO), *ζ*-carotene desaturase (ZDS), and carotenoid isomerase (CRTISO). Cyclization of lycopene under the action of *ɛ*-ring cyclase (LCYE) and/or *β*-ring cyclase (LCYB) is another important process in the pathway, generating orange *α*-carotene and *β*-carotene. These carotenes can further convert to various carotenoids, such as lutein, zeaxanthin, antheraxanthin, violaxanthin, and capsanthin, by *β*-ring hydroxylase (HYB), *ɛ*-ring hydroxylase (HYE), zeaxanthin epoxidase (ZEP), and capsanthin synthase (CCS) (Clotault et al., 2008). Recently, studies on the carotenoid-regulated leaf coloration mechanisms have been well characterized. For instance, the *A. thaliana im* variegation mutant has both green and white leaf sectors, the white leaf sectors have low carotenoid levels due to a deficiency in AtPSY enzymatic activity (Aluru et al., 2009). Li et al. (2018) found that upregulated expression of *GbZ-ISO*, *GbZDS*, and *GbLCYE* genes may increase carotenoid accumulation in the ginkgo (*Ginkgo biloba*) golden-green leaf mutant. Additionally, *PSY*, *PDS*, *ZDS*, and *LCYB* were successfully transferred into several plants, including rice, tomato, and potato (*Solanum tuberosum*), which enhanced carotenoid concentrations in transgenic plants (Rosati, Diretto & Giuliano, 2009).

Sweet osmanthus (*Osmanthus fragrans*) is an evergreen shrub or small tree species of the genus *Osmanthus* (Oleaceae) and is one of the top 10 traditional Chinese flowering plants due to its high ornamental value and unique sweet aroma (Shang, Yin & Xiang, 2003). Long-term hybridization and artificial selective breeding have led to the development of a large number of cultivars, which are divided mainly into four groups according to their phenotypic characteristics (*e.g.*, leaf shape, flower color, aroma, and flowering season, etc.): Asiaticus, Albus, Luteus, and Aurantiacus (Xiang & Liu, 2008). In recent years, many cultivars that produce colorful leaves have been cultivated in China. For example, the new cultivar ‘Qiannan Guifei’ (belonging to the Albus group) produces red buds, stems, and leaves. The leaves gradually lose their red color, turn white to yellow-green, and become completely green as they develop. The colorful leaves last for almost half a year. Therefore, these colored-leaf cultivars are a valuable germplasm resource that can be used to breed new cultivars. They also have high market value and potentially widespread applications in landscaping. However, the mechanisms underlying the leaf color transformation have not been elucidated.

Here, we studied the regulatory mechanism of leaf coloration in the new colored-leaf cultivar ‘Qiannan Guifei’. In particular, we compared chloroplast ultrastructure, pigment concentration, and transcriptomic data during the process of leaf color changes. We identified differentially expressed genes (DEGs) involved in the flavonoid, Chl and carotenoid metabolic pathways and further screened those DEGs responsible for the leaf coloration. Our results revealed the biochemical characteristics and molecular mechanisms underlying pigment-regulated leaf coloration and provide potential candidate genes for leaf color improvement *via* genetic modification in *O. fragrans*.

## Material and Methods

### Plant materials and sampling

**Figure 1:**
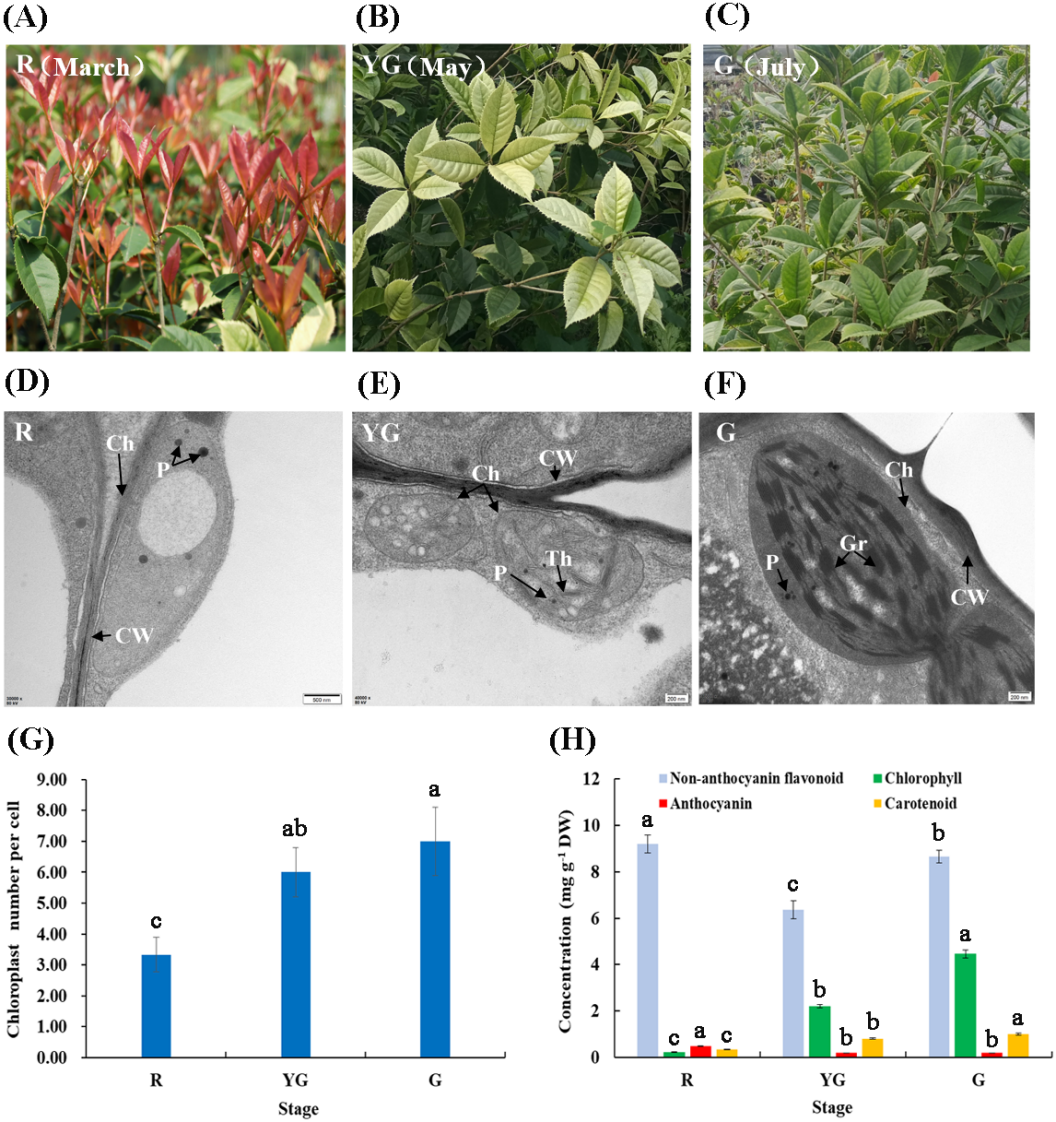
(A–F) Leaf characteristics and chloroplast ultrastructures of ‘Qiannan Guifei’ at the three developmental stages. (G–H) Average number of chloroplasts per cell and pigment concentrations at the three stages. Ch, Chloroplast; CW, Cell wall; Gr, Grana; P, Plastoglobuli; Th, Thylakoid. Bars represent the standard errors of three biological replicates. Different lowercase letters indicate a significant difference (*p* < 0.05) relative to the value at the G stage, as determined using ANOVA analysis, which is based on Duncan’s multiple range test.

Two-year-old ‘Qiannan Guifei’ plants were potted and grown under field conditions at Zhejiang Sci-Tech University, Zhejiang, China (30°16′N, 120°12′E). The development of new leaves of ‘Qiannan Guifei’ was divided into three different stages according to changes in leaf color (Figs. 1A–1C). In March and April, new shoots had red leaves, which was defined as the red (R) stage. As the leaves developed, the red color faded, and the new shoots produced yellow-green leaves from late April to June; this period was defined as the yellow-green (YG) stage. In July, leaves were completely green; this period was defined as the green (G) stage. The first pair of leaves from top of the shoots was collected separately at the R, YG, and G stages. Sample pools were constructed from 15 independent plants at each stage. These plants were divided into three groups to obtain three biological replicates. Thus, each replicate consisted of 10 leaves from five plants. In total, 30 leaves were harvested from each stage. Among them, nine fresh leaves from three biological replicates of each stage were used to evaluate chloroplast ultrastructure. Fifteen leaves from three biological replicates of each stage were used to measure the concentrations of different pigments. The remaining leaves were used to extract RNA. For pigment and RNA extraction experiments, the collected leaves were immediately frozen in liquid nitrogen and stored at −80 °C.

### Observation of chloroplast ultrastructure

Fresh leaves collected to examine chloroplast ultrastructure were cut into small sections (0.5 × 0.5 × 0.5 mm) along main vein and prefixed in 2.5% (v/v) glutaraldehyde for 24 h at 4 °C followed by 1% (v/v) osmium acid for 1–2 h. Samples were then dehydrated through an ethanol series, embedded in resin, cut into ultrathin sections, and stained with 1% (w/v) uranyl acetate and 1% (w/v) alkaline lead citrate. Leaf cells were subsequently observed and imaged using an H-7650 transmission electron microscope (Hitachi, Tokyo, Japan). Chloroplasts in 30 different cells were counted to calculate the average number of chloroplasts per cell.

### Measurements of non-anthocyanin flavonoid and anthocyanin concentrations

The non-anthocyanin flavonoid concentration was assayed according to the methods of Khanam et al. (2012). Freeze-dried leaves were first ground into powder. Then, 0.5 g of the powder was extracted with 15 mL of 80% methanol for 24 h and suspended by sonication for 30 min. After centrifugation, supernatant was passed through a 0.22 µm Millipore filter, and a two mL aliquot of the supernatant, two mL of 1.5% AlCl_3_, three mL of ddH_2_O, and three mL of 1 M acetic acid sodium acetate (pH 5.0) were mixed together for 10 min. Absorbance of the reaction mixture was determined at 415 nm on a BioMate 3S ultraviolet–visible spectrophotometer (Thermo Fisher Scientific, Shanghai, China). The anthocyanin concentration was determined according to the methods of Ray et al. (2003). Samples were ground into powder, heated to 55 °C for 10 min in 10 mL of 2 mol L^−1^ HCl, and then incubated overnight in the dark. Extract was clarified by centrifugation, and absorbance of the supernatant was measured at 523 nm using the ultraviolet spectrophotometer.

### Measurements of carotenoid concentration

The carotenoid concentration was measured according to the methods of Sarker & Oba (2018), with slight modifications. Freeze-dried leaves were ground, after which 0.5 g of leaf tissue was extracted with 15 mL petroleum ether at 4 °C in the dark for 24 h, and then extract was suspended by sonication for 30 min. After centrifugation, absorbance of the supernatant was determined at 440 nm on the ultraviolet spectrophotometer.

### Measurements of Chl concentration

The Chl concentration was determined according to the methods of Arnon (1949). Freeze-dried leaves collected at the different stages were ground into powder, after which 0.5 g of the powder was immersed in 80% acetone for 24 h at 4 °C in the dark to extract the Chl. Absorbance of the extract was measured at 663 nm and 645 nm using the ultraviolet spectrophotometer to determine the Chl a and Chl b concentrations, respectively. The total Chl concentration was the sum of Chl a and Chl b.

### RNA extraction, cDNA library construction, and Illumina deep sequencing

Total RNA was isolated from the leaves collected at the R, YG, and G stages using an RNAisomate RNA EasySpin Isolation System (Aidlab Biotech, Beijing, China) according to the manufacturer’s instructions. Three biological replicate samples were collected at each stage (nine samples in total). RNA quality was assessed *via* 1% agarose gel electrophoresis and verified using a NanoDrop 1000 spectrophotometer (NanoDrop, Wilmington, DE, USA). In addition, RNA integrity and concentration were checked using an Agilent 2100 Bioanalyzer (Agilent Technologies, CA, USA).

RNA fragmentation, cDNA synthesis, and PCR amplification processes were conducted according to the Illumina RNA-Sequencing (RNA-Seq) protocol. Equal amounts of RNA from each sample were used to construct a cDNA library. A total of nine cDNA libraries were sequenced using the Illumina BGISEQ-500 system at BGI Technology Corporation (BGI, Shenzhen, China). Low-quality reads were removed from raw reads using SOAPnuke v1.4.0 software (BGI, Shenzhen, China). Clean reads were then mapped to an *O. fragrans* reference genome (Yang et al., 2018) using HISAT v2.1.0 software (Kim, Langmead & Salzberg, 2015). The raw reads are available in NCBI Sequence Read Archive (SRA) under the accession number SRP255205. The assembled sequences (length >200 bp) have been deposited at DDBJ/ENA/GenBank under the accession number GJHS00000000. Other sequences (length <200 bp) are shown in Supplementary file 1. To evaluate repeatability of the three sequencing results for each stage, Pearson’s correlation analysis was conducted *via* COR function in R v.4.0.4 software (R Core Team, 2021).

### Identification and functional analyses of DEGs

Gene expression levels were determined in terms of fragments per transcript kilobase per million fragments mapped (FPKM) reads. Gene expression libraries corresponding to the three different stages were organized into three pairwise comparisons (R *vs* G, R *vs* YG, and YG *vs* G) to identify the DEGs. The DEGseq R package (Wang et al., 2010) was used to screen genes that were differentially expressed—those with corrected *p*-value ≤ 0.001 and —log_2_ ratio—≥ 1 (Dong, Xu & Ji, 2019). Biological functions of the DEGs were determined by subjecting them to Gene Ontology (GO) and Kyoto Encyclopedia of Genes and Genomes (KEGG) enrichment analysis. In addition, agriGO (http://bioinfo.cau.edu.cn/agriGO/) was used to test statistical enrichment of the DEGs in GO terms, and KOBAS 2.0 (http://kobas.cbi.pku.edu.cn/) was used to identify enriched KEGG pathways (http://www.genome.jp/kegg/). GO terms and KEGG pathways with Q-values ≤ 0.05 were considered significantly enriched categories. Bubble maps and heatmaps were constructed using OmicShare tools (http://www.omicshare.com/tools).

### Identification and phylogenetic analyses of *OfMYBs*

The putative *OfMYB* genes associated with flavonoids biosynthesis in ‘Qiannan Guifei’ leaves were identified after initially screening of the differentially expressed *OfMYBs* according to the criteria [log_2_(R/YG) ≥ 1 and log_2_(R/G) ≥ 1]. To categorize and survey the evolutionary relationships of *OfMYBs*, multiple sequences of the selected *OfMYBs* and 35 *MYBs* from other plant species were aligned using ClustalW, and a phylogenetic tree was then constructed using MEGA 6.0, with the maximum-likelihood method (Tamura et al., 2013). GenBank accession numbers of the *MYBs* used in the phylogenetic analyses are shown in Table S1.

### qRT-PCR analysis

To validate the results from RNA-Seq analysis, nine critical DEGs associated with pigment metabolism were selected for qRT-PCR. These genes included five flavonoid biosynthesis-related genes, *OfPAL*, *OfF3H*, *OfDFR*, *OfANS*, and *OfUGT*; two carotenoid biosynthesis-related genes, *OfHYB* and *OfZEP*; and two Chl biosynthesis-related genes, *OfPOR* and *OfCAO*. Extraction of total RNA from the samples and synthesis of first-stranded cDNA were conducted according to the manufacturer’s instructions. Synthesized cDNA was diluted with water (1:40), after which the diluted cDNA was used as a template for qRT-PCR. Primers were designed using Primer Premier 5.0 (Premier Biosoft, CA, USA). Primer sequences are shown in Table S2. qRT-PCR was performed in a reaction mixture with a total volume of 20 *μ*L. Each reaction mixture consisted of 2 *μ*L of cDNA template, 4 *μ*L of each primer, and 10 *μ*L of 2 × SYBR Premix Ex Taq, and the analysis was carried out with a Bio-Rad CFX96 Real-Time PCR System (Bio-Rad, Hercules, CA, USA) according to the manufacturer’s instructions. Amplification reactions involved an initial denaturation step at 95 °C for 1 min, followed by 40 cycles of 5 s at 95 °C, 30 s at 55 °C, and 30 s at 72 °C. Each reaction was carried out in triplicate. *OfActin* was used as a reference gene. Relative expression level of the selected gene was calculated using the 2^−ΔΔCt^ method against the expression level of internal control. Three biological replications were included for each gene.

### Statistical analysis

All the experiments were performed in accordance with a completely randomized design, and three biological replicates were included. For the measurements of different pigments, the absorbance value was normalized to the dry weight (DW) of each sample to indicate pigment concentrations. The pigment concentrations were subjected to one-way ANOVA followed by Duncan’s multiple range test in SPSS 20.0 software (IBM, IL, USA). Data were represented as the mean ±SD.

## Results

### Chloroplast ultrastructure and pigment concentrations

Chloroplast morphology and distribution in leaves of ‘Qiannan Guifei’ were compared during the process of leaf color changes. At the R stage, the chloroplasts had no intact membrane structures and contained large vesicles and small plastoglobuli (Fig. 1D). At the YG stage, some thylakoids were visible, but there were no stacks of grana (Fig. 1E). However, at the G stage, the chloroplasts had a typical structure with highly organized granum lamellae and distinct thylakoid membranes (Fig. 1F). Additionally, the average number of chloroplasts per cell at the YG and G stages was nearly double the number present at the R stage (Fig. 1G).

Pigment concentrations in leaves of ‘Qiannan Guifei’ were also measured at the three different stages. Fig. 1H shows that the anthocyanin concentration significantly increased to 0.48 mg g ^−1^ DW at the R stage compared to the G stage (0.19 mg g^−1^ DW), and similar levels were maintained between YG and G stages (0.19 mg g^−1^). The non-anthocyanin flavonoid concentration also increased at the R stage (9.19 mg g ^−1^ DW) compared to the G stage (8.64 mg g^−1^ DW), but a lower concentration was found at the YG stage (6.37 mg g^−1^ DW). Meanwhile, the carotenoid concentrations were 0.22 and 0.81 mg g^−1^ DW at the R and YG stages, respectively. These concentrations represent nearly 22.00% and 81.00% of that at the G stage (1.00 mg g ^−1^ DW). Similarly, the Chl concentration decreased to 0.33 and 2.20 mg g^−1^ DW at the R and YG stages, respectively, levels that were approximately 7.40% and 49.33% of that at the G stage (4.46 mg g^−1^ DW), respectively.

### Transcriptome sequencing and identification of DEGs

Approximately 55.08 Gb of clean bases were generated from nine cDNA libraries. After stringent quality checks, the number of clean reads ranged from 36,873,844 to 43,326,788 among all the libraries, and more than 80% of the clean reads could be mapped to the *O. fragrans* genome. All of the samples were of high quality, with an average Q30 of 90.27% (Table 1). In addition, a total of 30,598 novel coding transcripts, including coding transcripts, noncoding transcripts, novel isoforms, and novel genes, were obtained. The average length of transcript was 2,276 bp (ranging from 200 to 16,555; Tables S3–S4).

**Table 1:** Summary of transcriptome sequencing data.

Sample ID	Total raw reads	Total clean reads	Total clean bases/Gb	Total mapped	Uniquely mapped	Q30 (%)
R1	45167950	40717190	6.11	17677134 (86.83%)	10874651 (53.42%)	89.81
R2	48325832	42941952	6.44	18545367 (86.37%)	11563305 (53.86%)	90.34
R3	41257824	36873844	5.53	16084963 (87.24%)	9945731 (53.94%)	89.80
YG1	43841376	39082860	5.86	16777416 (85.86%)	10348844 (52.96%)	90.55
YG2	44825266	39865360	5.98	17117199 (85.88%)	10548862 (52.92%)	90.20
YG3	48416710	42738344	6.41	18394468 (86.08%)	11435965 (53.52%)	90.03
G1	49079576	43326788	6.50	18277109 (84.37%)	11421310 (52.72%)	91.06
G2	43114032	38479142	5.77	16286853 (84.65%)	9967791 (51.81%)	90.28
G3	48743488	43204494	6.48	17969148 (83.18%)	10982751 (50.84%)	90.40

**Figure 2:**
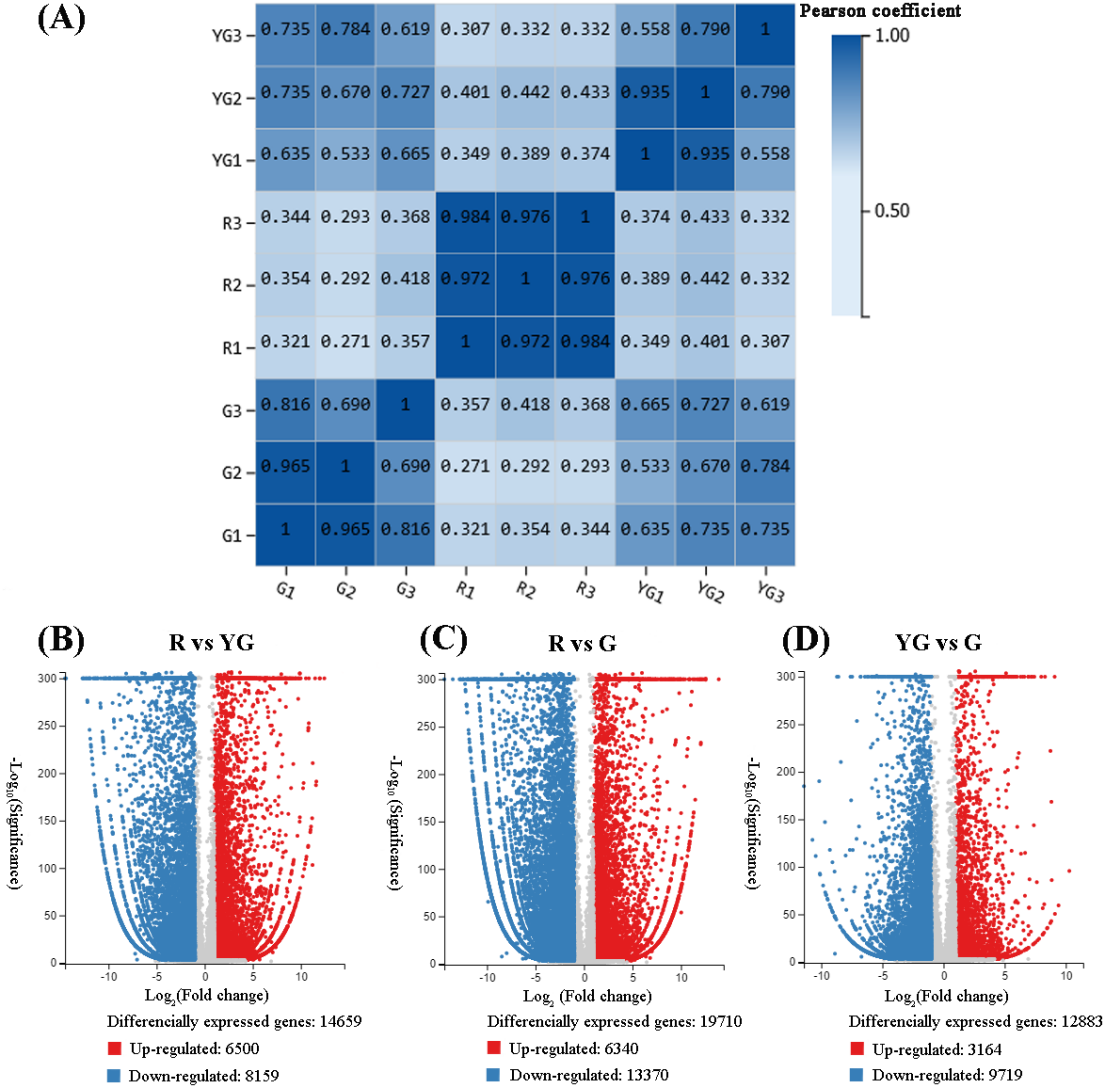
(A) Repeatability evaluation of transcriptome data from ‘Qiannan Guifei’ after using Pearson’s correlation analysis. (B–D) Volcano plot of DEGs among the three developmental stages. The upregulated and downregulated DEGs are shown in red and blue, respectively. The *x*-axis represents the fold changes on a log_2_ scale. The *y*-axis represents the negative −log_10_ transformed *p*-values (*p* < 0.05) of the *t*-test, which were used to identify differences among the three stages.

Correlations of sequencing results among the three replicates of samples from the R stage (*i.e.,* R1 *vs* R2, R1 *vs* R3, and R2 *vs* R3) were very high (*r* > 0.900). Similarly, pairwise correlations of YG1 *vs* YG2 and G1 *vs* G2 were also very high (*r* > 0.900). However, YG3 and G3 showed from low to moderate-high correlation (*r* < 0.816) with the other two replicates from the YG and G stages, respectively (Fig. 2A). Therefore, the YG3 and G3 samples were removed when DEGs were screened. As a result, 23,954 genes were identified as being significantly differentially expressed among the three stages (Figs. 2B–2D, Tables S5–S10). The greatest number of DEGs (19,710) occurred in the R *vs* G comparison, of which the expression of 6,340 was upregulated and 13,370 was downregulated. In the R *vs* YG comparison, 14,659 DEGs were obtained, including 6,500 upregulated and 8,159 downregulated DEGs. Meanwhile, 3,164 and 9,719 DEGs were upregulated and downregulated in the YG *vs* G comparison, respectively.

### Functional enrichment analysis of DEGs

To further elucidate the functional roles of significant DEGs during the process of leaf color changes, we performed GO enrichment and KEGG pathway enrichment analysis. The DEGs were significantly (Q-value ≤ 0.05) enriched in GO terms ‘amide biosynthetic process’ (GO: 0043604), ‘peptide biosynthetic process’ (GO: 0043043), ‘translation’ (GO: 0006412), and ‘structural molecule activity’ (GO: 0005198) in the R *vs* G and YG *vs* G comparisons (Tables S11–S12), whereas in the R *vs* YG comparison, most enriched DEGs were from the ‘protein kinase activity’ (GO: 0004672), ‘intrinsic component of membrane’ (GO: 0031224) and ‘movement of cell or subcellular component’ (GO: 0006928) categories (Tables S13). Furthermore, the top 20 KEGG pathways with high representation from R *vs* YG, R *vs* G, and YG *vs* G comparisons were identified. The KEGG terms associated with pigment metabolism, such as ‘phenylpropanoid biosynthesis’ (ko00940), ‘carotenoid biosynthesis’ (ko00906), ‘isoflavonoid biosynthesis’ (ko00943), ‘flavonoid biosynthesis’ (ko00941), and ‘anthocyanin biosynthesis’ (ko00942), were enriched in the R *vs* YG comparison (Table S14), whereas in the R *vs* G comparison (Table S15), the main enriched pigment metabolism pathway was ‘anthocyanin biosynthesis’. In addition, ‘porphyrin and chlorophyll metabolism’ (ko00860) and ‘anthocyanin biosynthesis’ were enriched in the YG *vs* G comparison (Table S16).

### DEGs involved in Chl metabolism pathway

**Figure 3:**
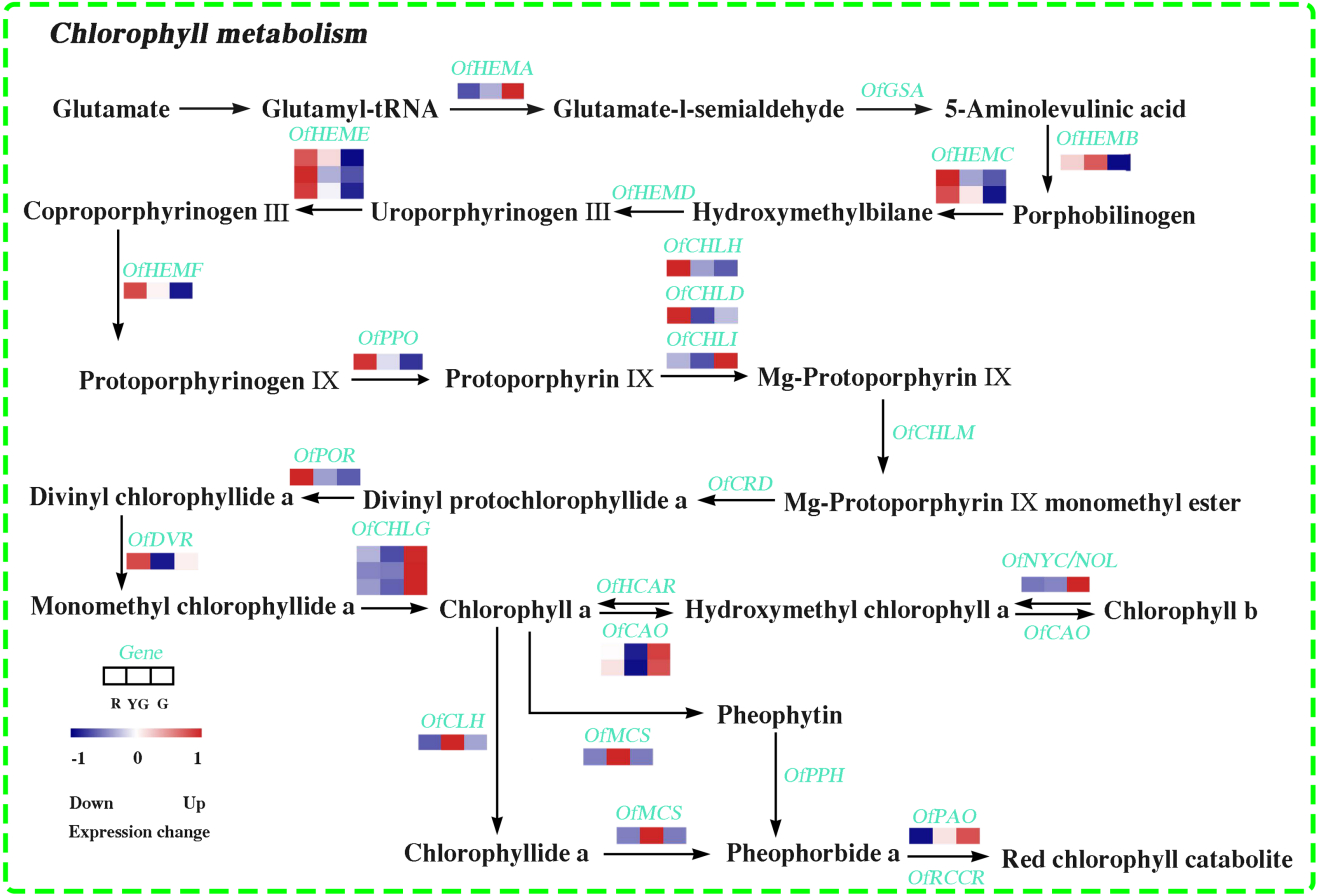
Expression profiles of the DEGs involved in chlorophyll metabolism at the three developmental stages. The scale bar represents the changes of gene expression pattern: red rectangle indicates upregulated expression pattern, blue rectangle indicates downregulated expression pattern, and the white rectangle indicates gene whose expression did not change. The normalized signal intensity ranged from −1.0 to 1.0, which was consistent with color changes from blue to red.

In this study, 22 DEGs involved in Chl biosynthesis and degradation were identified according to KEGG pathway assignment, and the expression profiles of these DEGs were analyzed *via* hierarchical clustering (Fig. 3 and Table S17). *OfHEMA* gene, encoding the first rate-limiting enzyme glutamyl-tRNA reductase in the Chl biosynthesis, was significantly upregulated from the R stage to the G stage. The expression levels of three *OfCHLG* genes and two *OfCAO* genes, which encode enzymes involved in the Chl a and Chl b biosynthesis, respectively, also continuously increased as the leaves developed. Similarly, *OfCHLI*, encoding a subunit of Mg-chelatase, presented higher transcript levels at the G stage. However, the other two subunits of Mg-chelatase, *OfCHLH* and *OfCHLD*, were expressed at higher levels at the R stage. In addition, four DEGs (*OfCLH*, *OfPAO*, *OfNOL/OfNYC*, and *OfMCS*) related to Chl a degradation showed two expression patterns during the three stages. The *OfPAO* and *OfNOL/OfNYC* transcript levels continuously increased from the R to the G stage, while *OfCLH* and *OfMCS* genes were highly expressed at the YG stage.

### DEGs involved in carotenoid biosynthesis pathway

**Figure 4:**
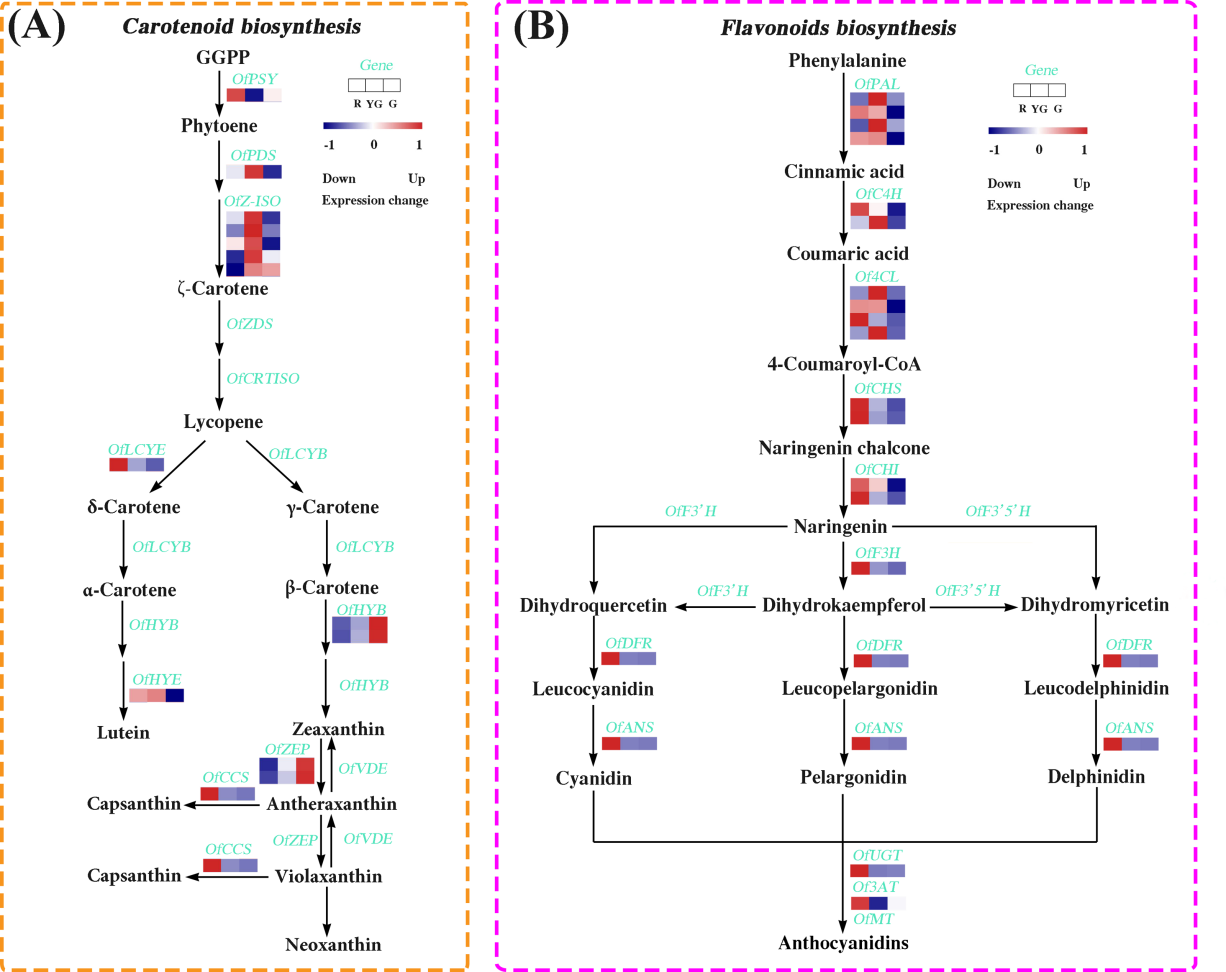
Expression profiles of the DEGs involved in (A) carotenoid and (B) flavonoid biosynthesis pathways at the three developmental stages. The colored rectangle indicates the gene expression pattern as defined in Fig. 3.

We identified 14 DEGs in the carotenoid biosynthesis pathway, the abundance of their transcripts varied among the different stages. The expression levels of the DEGs involved in *α*-carotene and *β*-carotene biosynthesis, such as *OfPSY*, *OfPDS*, *OfZ-ISO*, and *OfLCYE*, were much higher at the R or YG stage. Similarly, *OfCCS* and *OfHYE*, acting as the critical genes in capsanthin and zeaxanthin biosynthesis, respectively, were highly expressed at the R or YG stage. Additionally, *OfHYB* and *OfZEP*, which play important roles in the biosynthesis of zeaxanthin, antheraxanthin, and violaxanthin, exhibited increased expression patterns from the R to the G stage (Fig. 4A and Table S18).

### DEGs involved in flavonoid biosynthesis pathway

We also screened 19 DEGs associated with the flavonoid biosynthesis (Fig. 4B and Table S19). Among them, several DEGs involved in the early flavonoid biosynthesis pathway, such as *OfPAL*, *OfC4H*, and *Of4CL*, accumulated more transcripts at the R stage or YG stage, while those DEGs related to the late biosynthesis pathway, such as *OfCHS*, *OfCHI*, *OfF3H*, *OfDFR*, *OfANS*, *OfUGT* and *Of3AT*, showed the highest expression levels at the R stage.

### Differentially expressed *TFs*

**Figure 5:**
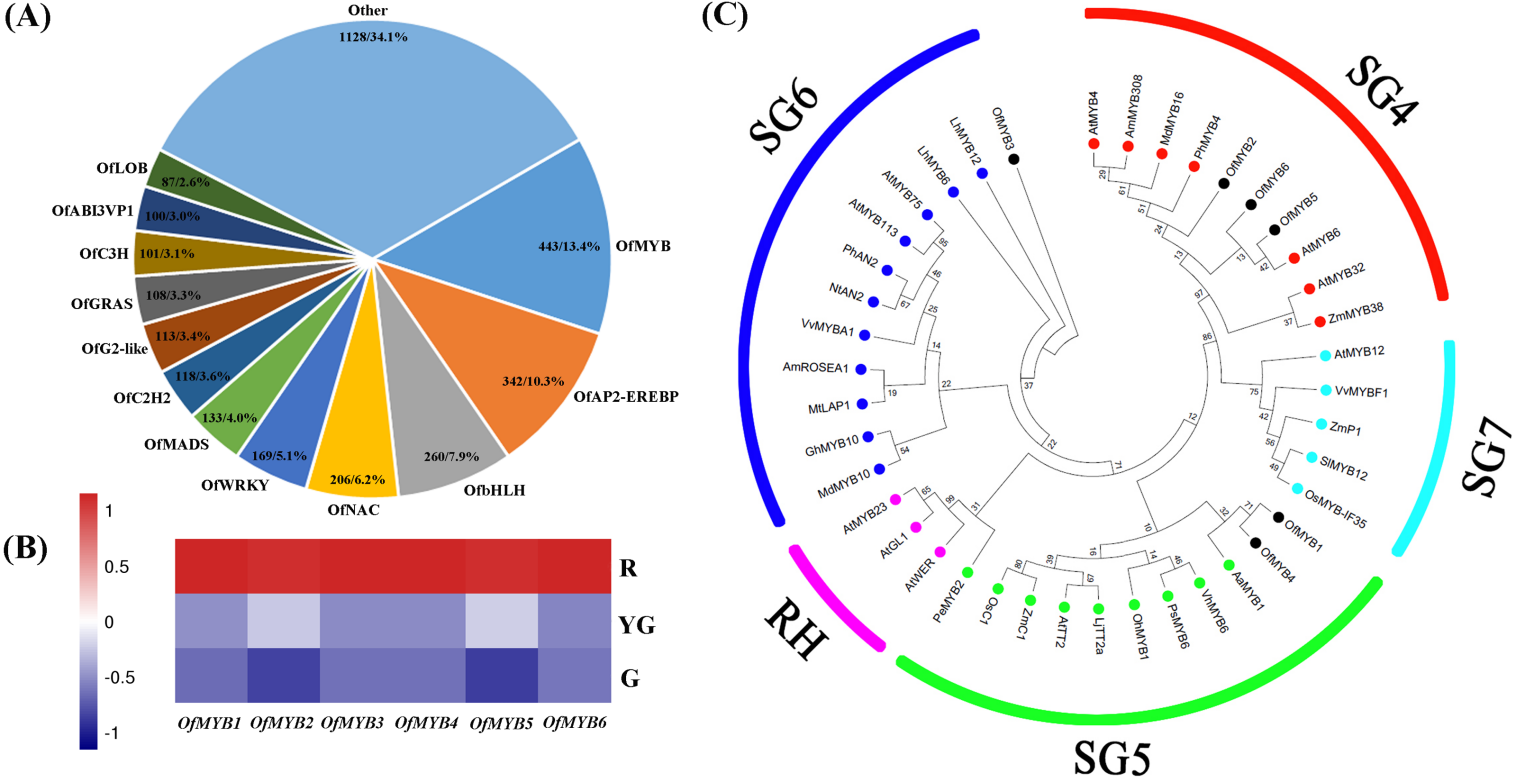
(A) Differentially expressed *TFs*. (B) Expression profiles of six differentially expressed *OfMYBs* at the three stages. (C) Phylogenic analysis of six *OfMYBs* with 35 flavonoid-related *MYBs* from other species. Full-length amino acid sequences from these*MYBs* were analyzed under maximum-likelihood (ML) phylogenetic methods. Numbers near branches indicate bootstrap values that were calculated from 1,000 replicates. *OfMYBs* are highlighted with solid black circles. *MYBs* phylogenetic tree contained five subgroups (SGs), SG4 (repressors of flavonoid biosynthesis), SG5 (activators of anthocyanin biosynthesis), SG6 (activators of anthocyanin biosynthesis), SG7 (activators of flavonol/flavone biosynthesis), and RH (activators of root hair growth). The subgroup of RH was included as an outgroup.

A total of 3,308 DEGs belonging to 59 TF families were screened in our data. *OfMYB* family members were the most abundant (443, 13.4%), followed by *OfAP2-EREBP* (342, 10.3%), *OfbHLH* (260, 7.9%), *OfNAC* (206, 6.2%), and *OfWRKY* (169, 5.1%) members (Fig. 5A). In flavonoid and anthocyanin biosynthesis, the spatial and temporal expression of several important structural genes, such as *C4H*, *F3H*, *DFR*, and *ANS*, is often regulated by MYB TFs (Yamagishi et al., 2010; Colquhoun et al., 2011; Starkevic et al., 2020). Based on the analyses of *OfMYBs* gene expression and their phylogenetic relationships with 35 *MYBs* from other species that are known to be involved in the accumulation of flavonoids (Schwinn et al., 2016; Cho et al., 2016; Zhou et al., 2016; Li et al., 2016b; Feng et al., 2018), we identified six *OfMYBs* (termed *OfMYB1–OfMYB6*) that might play important roles in regulating the flavonoid biosynthesis in ‘Qiannan Guifei’ red leaves. The transcript abundances of these *OfMYBs* were greatest at the R stage (Fig. 5B and Table S20). Phylogenetic analysis showed that the candidate *OfMYBs* were clustered together with known functional homologs from the SG4, SG5, and SG6 groups (Fig. 5C). In the SG4 group, *OfMYB5* and *OfMYB6* were closely related to *AtMYB6*, while *OfMYB2* was similar to *PhMYB4*. In the SG5 group, *OfMYB1* and *OfMYB4* clustered together with *AaMYB1*. In addition, *OfMYB3* was phylogenetically related to *LhMYB12* from the SG6 group.

### Validation by qRT-PCR

**Figure 6:**
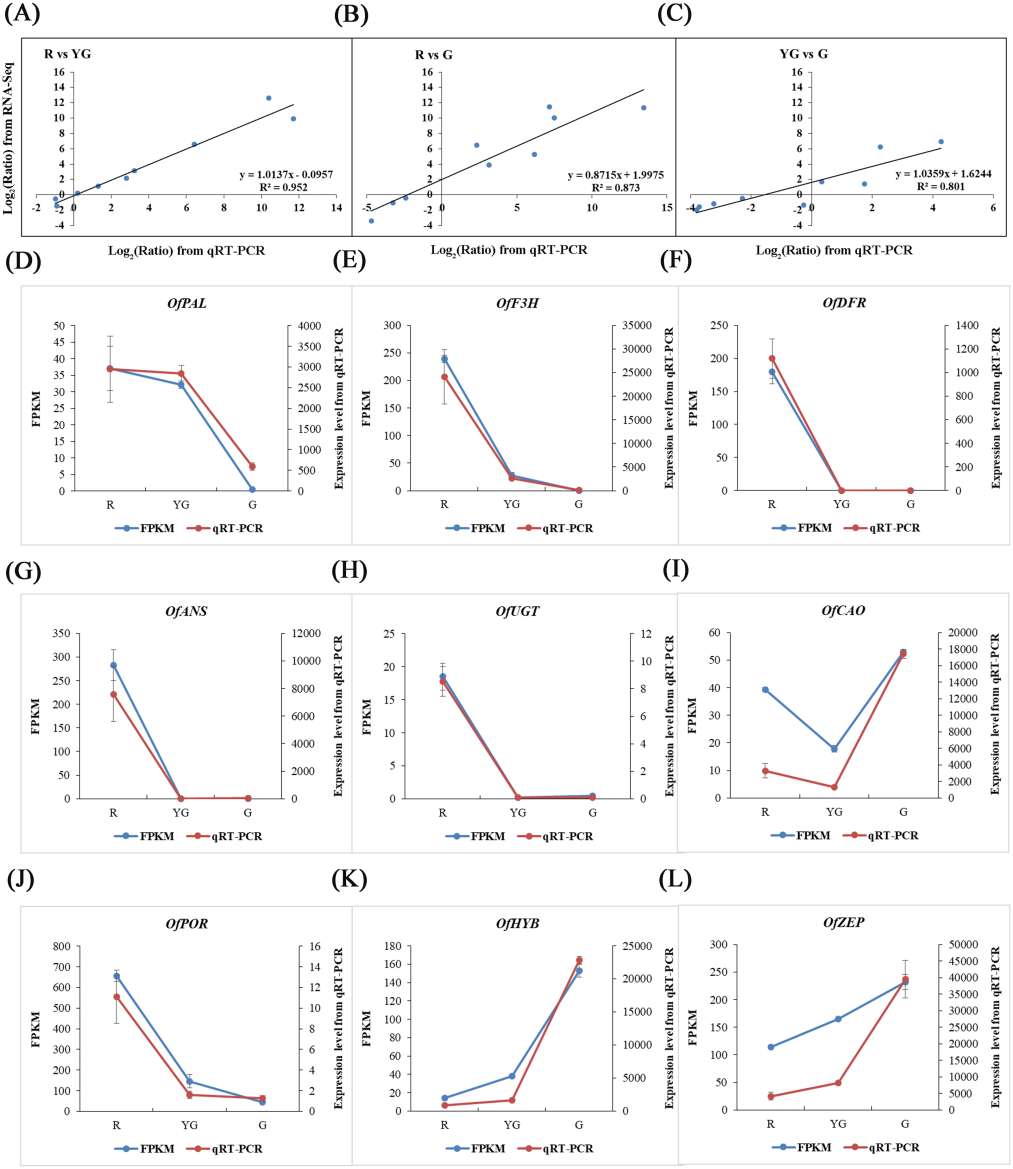
Validation of RNA-Seq results *via* qRT-PCR assays. Nine DEGs were selected from the flavonoid, carotenoid, and chlorophyll metabolic pathways. (A–C) Scatter diagrams show the correlations among expression levels of the nine DEGs as measured by the qRT-PCR (*x*-axis) and RNA-Seq (*y*-axis) analyses. The fold changes for gene expression were transformed as log _2_(R/YG), log _2_ (R/G), and log _2_(YG/G) from the RNA-Seq and qRT-PCR data. (D–L) Expression profiles of the nine DEGs revealed by RNA-Seq (left *y*-axis) and qRT-PCR (right *y*-axis) at the three stages. Expression levels obtained from the qRT-PCR were normalized to the reference gene *OfActin*.

The reliability of RNA-Seq data was validated by conducting a qRT-PCR-based differential expression analysis of the identified DEGs at the three stages. Linear regression analysis showed that the expression levels of nine pigment-related DEGs detected by qRT-PCR were positively correlated with those obtained from the RNA-Seq data (R^2^>0.800; Figs. 6A–6C), and all of the DEGs showed expression patterns in the qRT-PCR analysis that were consistent with the RNA-Seq data (Figs. 6D–6L), which verified the accuracy of our RNA-Seq results.

## Discussion

Development of plant organ color is often associated with some common trends. For example, maple (*Acer* spp.) leaves and numerous fruits are red in color during the late developmental stage. This is due to the breakdown of Chl, and large amounts of anthocyanins subsequently accumulate (Feild, Lee & Holbrook, 2001). However, different trends have been observed in leaves of *O. fragrans*, *e.g.*, the young and mature leaves of ‘Qiannan Guifei’ are red and green, respectively. Thus, this cultivar is a valuable material with which to explore specific biological processes that regulate leaf color transformation. In this study, we focused on determining the regulatory mechanism governing leaf color formation at three leaf developmental stages (R, YG, and G) in ‘Qiannan Guifei’ plants. Chloroplast ultrastructure and pigment concentrations differed considerably among the three stages. Transcriptional sequence analysis identified 61 DEGs associated with flavonoid, Chl, and carotenoid metabolisms. In addition, qRT-PCR validated the altered expression levels of these DEGs. Analyses of the expression patterns of pigment-related DEGs confirmed the critical genes potentially responsible for leaf coloration in ‘Qiannan Guifei’.

### Regulation of flavonoid-related genes in ‘Qiannan Guifei’ leaves

Anthocyanins are the final product of flavonoid biosynthesis and play important roles in red and purple leaf coloration in plants (Kerio et al., 2012; Anderson & Ryser, 2015); (Lo Piccolo et al., 2020). In this study, anthocyanin concentrations decreased according to leaf color change from red to green in ‘Qiannan Guifei’ leaves. At the R stage, the concentrations were approximately 2.5 times higher than those at the YG and G stages. Non-anthocyanin flavonoid concentrations were also the highest at the R stage. The abundance of flavonoids, especially anthocyanins, might mask the coloration of other pigments in ‘Qiannan Guifei’ leaves, leading to the appearance of a red color at the R stage.

As expected, increasing accumulation of anthocyanins in red leaves was correlated with increased expression of multiple structural genes involved in the flavonoid pathway. We found that seven structural genes in this pathway were expressed at the highest level at the R stage (Fig. 4). Chalcone synthase (CHS) participates in the first reaction of anthocyanin biosynthesis and is responsible for generating chalcone, which is a critical precursor substance for anthocyanin and other flavonoid biosynthesis, whereas CHI subsequently catalyzes the conversion of chalcone to naringenin (Heredia, Heredia-Guerrero & Dominguez, 2015; Lim & Li, 2017). Thus, if the expression levels of *CHS* and *CHI* are altered, the production of anthocyanins may also be affected accordingly. A previous study showed that silencing of the *ThCHS* gene could be used to modulate flower color from blue to white in torenia (*Torenia hybrida*) (Fukusaki et al., 2004). Halbwirth et al. (2006) reported that an increase in FaCHI enzyme activity caused elevated anthocyanin production during strawberry (*Fragaria* ×*ananassa*) fruit ripening. In the present study, *OfCHS* and *OfCHI* accumulated more transcripts at the R stage, suggesting that more chalcone was converted into naringenin in red leaves of ‘Qiannan Guifei’. F3H catalyzes the conversion of naringenin to yield dihydrokaempferol, and dihydrokaempferol can be converted to leucopelargonidin in a reaction catalyzed by DFR, after which colored pelargonidin compounds are generated under the action of ANS (Himi & Taketa, 2015). In the present study, *OfF3H*, *OfDFR*, and *OfANS* were highly expressed at the R stage, suggesting that pelargonidin might be closely related to the red coloration of ‘Qiannan Guifei’ leaves and that *OfF3H*, *OfDFR*, and *OfANS* play critical roles in regulating the accumulation of pelargonidin during this process.

During the last biosynthesis steps, glycosylation and acylation of anthocyanins are catalyzed by UGTs and ATs, respectively, which contributes to the generation of anthocyanin stable products (Tasaki et al., 2019). Lee et al. (2017) demonstrated that overexpression of *AtUGT* effectively promoted anthocyanin production in transgenic plants. In purple-red leaves of grape (*Vitis vinifera*), anthocyanin accumulation was found to be associated with the activation of *Vv3AT* and *VvUGT* (Matus et al., 2017). We found that the expression of *OfUGT* and *Of3AT* was significantly higher at the R stage, suggesting that the expression of both genes could accelerate the biosynthesis of stable anthocyanins in red leaves of ‘Qiannan Guifei’. However, several early flavonoid biosynthesis genes encoding OfPAL, OfC4H, and Of4CL, which catalyze important enzymatic reactions and provide precursors for flavones, flavonols, anthocyanins, and lignins (Mouradov & Spangenberg, 2014), exhibited relatively low expression levels at the R stage. A similar result was also observed in purple-leaf tea plants by Wei et al. (2016), who found that the expression levels of early flavonoid biosynthesis genes were less affected or not affected in purple buds and leaves than in green tissues, and most of the highly upregulated DEGs were identified as late biosynthesis genes. Thus, *OfPAL*, *OfC4H*, and *Of4CL* genes might not be critical genes affecting red coloration in ‘Qiannan Guifei’ leaves.

The regulation of flavonoid biosynthesis is controlled by the MBW complex, which consists of R2R3-MYB and basic helix-loop-helix (bHLH) TFs and a WD-repeat (WDR) protein (Nemesio-Gorriz et al., 2017). In this study, we screened six *OfMYB* s whose expression levels were highest at the R stage (Fig. 5B). Further analysis showed that these genes clustered together with *MYBs* related to flavonoid biosynthesis in other species (Fig. 5C). Among them, *OfMYB1/4* and *OfMYB3* are closely related to *AaMYB1* and *LhMYB12*, respectively. In previous studies, *AaMYB1* from anthurium (*Anthurium andraeanum*) was shown to be capable of activating anthocyanin production in the petals of an orchid (*Cymbidium* spp.) cultivar (Albert et al., 2010). *LhMYB12* determined organ- and tissue-specific accumulation of anthocyanins in a lily (*Lilium* spp.) cultivar (Yamagishi et al., 2010). Therefore, these homologous *OfMYBs* might play positive roles during the process of ‘Qiannan Guifei’ red leaf pigmentation. In contrast, three potential flavonoid-related *MYB* repressors, *OfMYB2* (a homolog of *P. hybrida PhMYB4*), *OfMYB5*, and *OfMYB6* (homologs of *AtMYB6*), were also identified. *AtMYB6* (Rowan et al., 2009) and *PhMYB4* (Colquhoun et al., 2011) have been demonstrated to possess strong repressive effects on flavonoid pathway genes. *PpMYB20*, which is also a homolog of *AtMYB6*, was found to be highly expressed with several anthocyanin-related *PpMYB* activators during peach (*Prunus persica*) petal development (Zhou et al., 2016). Taken together, these results suggested that flavonoids accumulation in ‘Qiannan Guifei’ red leaves may be controlled by a set of *OfMYB* genes, which includes at least three putative activators (*OfMYB1*, *OfMYB3*, and *OfMYB4*) and three putative repressors (*OfMYB2*, *OfMYB5*, and *OfMYB6*). Future research will focus on investigating the biological functions of these important *OfMYB* s and potentially revealing how these genes regulate flavonoids biosynthesis in *O. fragrans* leaves.

### Chloroplast development and regulation of Chl-related genes in ‘Qiannan Guifei’ leaves

Chloroplast development and Chl biosynthesis largely determine green leaf coloration. In chloroplasts, thylakoids are often arranged regularly and stacked into grana, which promotes the efficient conversion of solar energy to stored chemical energy (Zanini et al., 2021). Previous studies have demonstrated that some colored leaves display changes in the structural characteristics of chloroplasts. For instance, in *A. andraeanum*, the chloroplasts in green leaf tissues have a typical structure that involves many regular thylakoids, but the rubescent mutant contained incomplete thylakoid structures and was filled with irregularly arranged vesicles (Yang et al., 2015). Similarly, Shi et al. (2017) reported that mesophyll cells in a wheat (*Triticum aestivum*) albino mutant contained few chloroplasts, and the abnormal chloroplasts contained disordered granum lamellae. Our results showed that the chloroplasts in the leaves from the R and YG stages did not have an intact inner membrane structure and lacked grana, and the number of chloroplasts had decreased compared to that from the G stage, which indicated that abnormal chloroplast development had occurred during these stages (Figs. 1D–1F). In addition, the pigment measurement results showed that Chl concentration was significantly higher at the G stage (4.46 mg g^−1^ DW) than at the R (0.22 mg g^−1^ DW) and YG (2.20 mg g^−1^ DW) stages. Therefore, we speculated that aberrant chloroplasts might affect development in red and yellow-green leaves of ‘Qiannan Guifei’, leading to the restraint of Chl biosynthesis and leaf color variation.

Changes in any gene transcription during the Chl metabolism pathway likely influence the Chl level. Chl biosynthesis/degradation-related genes have been well elucidated in various species (Nagata et al., 2005). HEMA, which reduces the glutamyl moiety of glutamyl-tRNA to glutamate-1-semialdehyde, is the first enzyme regulating Chl synthesis. Studies have demonstrated that the Chl concentration was significantly decreased in *A. thaliana* under *AtHEMA* gene silencing (Kumar & Söll, 2000). *CHLG* and *CAO* are key genes regulating Chl a and Chl b production, respectively (Shalygo et al., 2009). Shen et al. (2020) demonstrated that silencing *HvCHLG* gene caused leaf yellowing in *H. vulgare*. In contrast, ectopic expression of *AtCAO* resulted in an increased abundance of light-harvesting Chl proteins in transgenic tobacco (*Nicotiana tabacum*) plants (Biswal et al., 2012). In our study, the transcript levels of *Of HEMA*, *OfCHLG*, and *OfCAO*, as well as the Chl concentrations, were significantly lower at the R and YG stages than at the G stage. Our results suggested that the low expression of these genes caused a reduction in enzyme activity and affected Chl biosynthesis in red and yellow-green leaves of ‘Qiannan Guifei’.

Some studies have demonstrated that the Chl biosynthesis relies on active Mg-chelatase, and Chl-deficient mutants are largely due to a lack of Mg-chelatase. Mutation of Mg-chelatase subunits CHLI, CHLD, or CHLH can influence its enzymatic activity, thus causing Chl metabolic disorders (Meier et al., 2011). Hansson et al. (2002) found that the decreased Chl levels in chlorotic seedlings of *H. vulgare* were caused mainly by a limited number of functional HvCHLI-HvCHLD-HvCHLH complexes. Campbell et al. (2015) also reported that no transcript of *GmCHLI* gene was detected in a chlorotic-leaf mutant of soybean (*Glycine max*). In the present study, the expression of *OfCHLH* and *OfCHLD* was dramatically decreased during the process of leaf color changes, while that of *OfCHLI* was increased. This differential expression pattern might affect the generation of Mg-protoporphyrin IX and ultimately reduce Chl biosynthesis in red and yellow-green leaves of ‘Qiannan Guifei’.

Development of colored leaves from the Chl-deficient mutants is also related to Chl degradation. CLH has long been considered the rate-limiting enzyme during the process of Chl degradation. It catalyzes the breakdown of Chl a into chlorophyllide a, followed by the removal of Mg^2+^ from the substrate with the involvement of MCS. Chl catabolites are then produced by the catalysis of PAO (Hoertensteiner, 2013). Previous studies have suggested that *ClCLH* is involved in Chl degradation in ethylene-treated lemon (*Citrus limon*) fruits (Shemer et al., 2008). In *V. vinifera* fruits, increased *VvMCS* transcript abundance is negatively correlated with Chl a concentration (Silva-Sanzana et al., 2016). We found that the expression levels of *OfCLH* and *OfMCS* were the highest at the YG stage. Both of these genes may have caused Chl a to degrade more rapidly in yellow-green leaves. PAO is also responsible for Chl a degradation, while NOL/NYC participates in the decomposition of Chl b to hydroxymethyl Chl a (Kusaba et al., 2007). Our results demonstrated that the expression of *OfPAO* and *OfNOL*/*OfNYC* tended to increase from the R stage to the G stage. However, the Chl concentration also increased during this process. Similar results were found in tea (Li et al., 2016a), probably due to the posttranscriptional regulation of *OfPAO* and *OfNOL*/*OfNYC* restricting enzyme bioactivity or the Chl degradation rate being slower than its rate of biosynthesis.

### Regulation of carotenoid-related genes in ‘Qiannan Guifei’ leaves

Carotenoids are synthesized in various plant plastids but are maintained at high levels in the chloroplast, where they can form photosynthetic complexes (Rezaei, Deokar & Tar’an, 2016). In this study, pigment analysis revealed that carotenoid concentrations were significantly higher at the G (1.0 mg g^−1^ DW) than at the R (0.33 mg g^−1^ DW) and YG (0.81 mg g^−1^DW) stages (Fig. 1H). Together with the structural characteristics of chloroplasts, our results suggested that granum deficiency not only blocked Chl biosynthesis in colored leaves of ‘Qiannan Guifei’ plants but also inhibited carotenoid biosynthesis.

Changes in carotenoid accumulation are associated with the expression of carotenoid biosynthesis-related genes. PSY is the first rate-limiting enzyme in the carotenoid biosynthesis pathway, and it directly promotes the biosynthesis of phytoene. Phytoene is converted to *ζ*-carotene by PDS and Z-ISO (Lindgren, Stalberg & Hoglund, 2003). Previous studies found that overexpression of the *ZmPSY* gene from *Z. mays* contributed to higher carotenoid levels in *O. sativa* endosperm (Paine et al., 2005). In contrast, silencing of the *PSY* (Kato et al., 2017) or *PDS* (Srinivasan, Babu & Gothandam, 2017) gene caused carotenoid deficiency in plants. Additionally, *OfPSY*, *OfPDS*, and *OfZ-ISO* were identified as the key genes affecting carotenoid accumulation during flower bud development in an orange-red-flowered cultivar of *O. fragrans* (Zhang et al., 2016). We found that the *OfPSY* gene was expressed at the highest level at the R stage and then showed a downregulated expression pattern, *OfPDS* and *OfZ-ISO* reached the highest expression levels at the YG stage, while carotenoid concentration continued to increase from the R stage to the G stage. It seems that *OfPSY*, *OfPDS*, and *OfZ-ISO* may play a small role in the carotenoid accumulation of ‘Qiannan Guifei’ leaves.

LCYB and LCYE are closely related to the generation of *α*-carotene and *β*-carotene, while HYE, HYB, ZEP, and CCS are involved in the biosynthesis of other carotenoids (Kim et al., 2012). Overexpression or knockout of *DcLCYB* in carrot (*Daucus carota*) effectively promoted or suppressed carotenoid levels (Moreno et al., 2013). Similarly, high expression levels of *FaLCYE* and *FaHYE* were found to be related to carotenoid accumulation in *F.* ×*ananassa* fruits (Zhu et al., 2015b). Yamamizo, Kishimoto & Ohmiya (2010) also found that morning glory (*Ipomoea nil*) plants lacked the ability to synthesize carotenoids, mainly resulting from the downregulation of *InHYB* and *InZEP* expression. In addition, Jeknic et al. (2012) showed that overexpression of *LlCCS* gene from tiger lily (*Lilium lancifolium*) caused the transgenic callus to accumulate more carotenoids, and the callus color changed from yellow to red-orange. In the present study, *OfHYB* and *OfZEP* were upregulated continuously, which was consistent with the changes of carotenoid levels, suggesting that both genes are closely related to carotenoid production during the process of leaf color changes. However, we did not identify any DEGs for *OfLCYB*, it was probably not expressed during the three stages or was expressed at very low levels that could not be detected. The expression of *OfLCYE* and *OfCCS* decreased continuously from the R to G stage, while the expression of *OfHYE* increased from the R to YG stage and then significantly decreased at the G stage. These different gene expression patterns suggested the presence of the extraordinary complexity of carotenoid metabolism underlying ‘Qiannan Guifei’ leaf color transformation.

## Conclusions

In this study, we compared red, yellow-green and green leaves from *O. fragrans* ‘Qiannan Guifei’ at the biochemical and transcriptomic levels. Lower Chl concentration and abnormal chloroplast structure might affect red and yellow-green leaf coloration, while higher flavonoid concentrations, especially anthocyanins, directly contribute to red leaf coloration. We found a total of seven structural genes (*OfCHS*, *OfCHI*, *OfF3H*, *OfDFR*, *OfANS*, *OfUGT*, and *Of3AT*) that were highly expressed in red leaves, promoting the biosynthesis of flavonoids, especially anthocyanins. The accumulation of flavonoids, including anthocyanins, may be regulated by three putative *MYB* activators (*OfMYB1*, *OfMYB3*, and *OfMYB4*) and three putative *MYB* repressors (*OfMYB2*, *OfMYB5*, and *OfMYB6*). In addition, we found that the expression of DEGs associated with Chl biosynthesis (*OfHEMA*, *OfCHLG*, and *OfCAO*) and carotenoid biosynthesis (*OfHYB* and OfZ*EP*) was continuously upregulated as leaves developed, indicating that these genes play important roles in the production of Chl and carotenoids, respectively. Our results provide a foundation for fully elucidating the regulatory mechanisms underlying leaf coloration in ‘Qiannan Guifei’ and highlight potential candidate genes that could be used in the molecular breeding of colored-leaf cultivars.

## Supplemental Information

10.7717/peerj.12265/supp-1 Supplemental Information 1 Six *OfMYBs* and 35 *MYBs* from other plant species used for phylogenetic analysis

10.7717/peerj.12265/supp-2 Supplemental Information 2 Table S2 Primers used for qRT-PCR.
Table S3 Summary of novel transcript.
Table S4 Summary of assembly quality of novel coding transcript.

10.7717/peerj.12265/supp-3 Supplemental Information 3 Table S5 Up-regulated DEGs in R vs YG group.
Table S6 Down-regulated DEGs in R vs YG group.
Table S7 Up-regulated DEGs in R vs G group.
Table S8 Down-regulated DEGs in R vs G group.
Table S9 Up-regulated DEGs in YG vs G group.
Table S10 Down-regulated DEG

10.7717/peerj.12265/supp-4 Supplemental Information 4 Table S11 Top 20 enriched GO terms in R vs G group.
Table S12 Top 20 enriched GO terms in YG vs G group.
Table S13 Top 20 enriched GO terms in R vs YG group.

10.7717/peerj.12265/supp-5 Supplemental Information 5 Table S14 Top 20 enriched pathways in R vs YG group.
Table S15 Top 20 enriched pathways in R vs G group.
Table S16 Top 20 enriched pathways in YG vs G group.

10.7717/peerj.12265/supp-6 Supplemental Information 6 Table S17 DEGs involved in chlorophyll metabolism during the leaf color change process

10.7717/peerj.12265/supp-7 Supplemental Information 7 Table S18 DEGs involved in carotenoid biosynthesis during the leaf color change process

10.7717/peerj.12265/supp-8 Supplemental Information 8 Table S19 DEGs involved in flavonoid biosynthesis during the leaf color change process

10.7717/peerj.12265/supp-9 Supplemental Information 9 Table S20 Six differentially expressed *OfMYBs.*

10.7717/peerj.12265/supp-10 Supplemental Information 10 The assembled sequences (length <200 bp) of *Osmanthus fragrans*

## Additional Information and Declarations

### DNA Deposition

The following information was supplied regarding the deposition of DNA sequences: The raw RNA-Seq reads are available at the NCBI Sequence Read Archive (SRA): SRP255205. The assembled sequences (length >200 bp) are available at DDBJ/ENA/GenBank under the accession number GJHS00000000
The DEGs used in phylogenetic and qRT-PCR analyses are available at GenBank: MT481993 (OfActin), MT481994 (OfMYB1), MT481995 (OfMYB2), MT481996 (OfMYB3), MT481997 (OfMYB4), MT481998 (OfMYB5), MT481999 (OfMYB6), MT495415 (OfANS), MT495416 (OfCAO), MT495417 (OfDFR), MT495418 (OfF3H), MT495419 (OfHYB), MT495420 (OfPAL), MT495421 (OfPOR), MT495422 (OfUGT), and MT495423 (OfZEP).

### Data Availability

The following information was supplied regarding data availability: The raw data (assembled sequences (length >200 bp) of Osmanthus fragrans) are available in the Supplementary File.
